# Relationship between Improvement in Physical Activity and Three Nutritional Assessment Indicators in Patients Admitted to a Convalescent Rehabilitation Ward

**DOI:** 10.3390/nu16152531

**Published:** 2024-08-02

**Authors:** Yusuke Tamamura, Chihiro Hachiuma, Michiko Matsuura, Sumiko Shiba, Toshio Nishikimi

**Affiliations:** 1 Department of Rehabilitation, Wakakusa-Tatsuma Rehabilitation Hospital, 1580 Ooaza Tatsuma, Daito 574-0012, Osaka, Japan; yt.tatsuma3586@gmail.com (Y.T.); wakatatsu.mmp@gmail.com (M.M.); 2 Department of Nutrition, Wakakusa-Tatsuma Rehabilitation Hospital, 1580 Ooaza Tatsuma, Daito 574-0012, Osaka, Japan; 3 Department of Physical Therapy, Konan Women’s University, 6-2-23 Morikita-cho, Higashinada-ku, Kobe 658-0001, Hyogo, Japan; s.shiba@konan-wu.ac.jp; 4 Department of Medicine, Wakakusa-Tatsuma Rehabilitation Hospital, 1580 Ooaza Tatsuma, Daito 574-0012, Osaka, Japan

**Keywords:** nutrition, physical activity, rehabilitation, convalescent rehabilitation wards

## Abstract

We investigated the relationship between three nutritional indicators, the Mini Nutritional Assessment-Short Form (MNA-SF), Geriatric Nutritional Risk Index (GNRI), and Controlling Nutrition Status (CONUT), and physical activity at discharge in patients admitted to convalescent rehabilitation wards. The study included 1601 patients (77 ± 12 years, male 46.2%) discharged from convalescent rehabilitation wards between April 2018 and September 2023. MNA-SF, GNRI, and CONUT scores were obtained on admission. Patients were divided into two groups according to their level of Functional Independence Measure (FIM) walk score at discharge. The walking group (n = 1181, FIM walk score ≥ 5, 76 ± 13 years, male 47.2%) was significantly younger than the wheelchair group (n = 420, 79 ± 12 years, FIM walk score < 5, male 43.8%) and had significantly higher MNA-SF (6.5 ± 2.5 vs. 4.7 ± 2.4) and GNRI (93.1 ± 12.4 vs. 86.7 ± 10.9) scores and significantly lower CONUT (3.1 ± 2.3 vs. 3.9 ± 2.3) scores than the wheelchair group (all *p* < 0.01). Multivariate logistic regression analysis showed that age, handgrip strength, Functional Oral Intake Scale, and MNA-SF score were independently associated with walking ability at discharge (all *p* < 0.01). In addition, MNA-SF scores were independently associated with Rehabilitation Effectiveness. These results suggest that nutritional status, particularly MNA-SF scores on admission, is associated with improvement of physical activity at discharge.

## 1. Introduction

Malnutrition is one of the most relevant conditions adversely affecting the health of the elderly [1], and the prevalence of malnutrition is high in convalescent rehabilitation wards with large elderly populations [2]. Indeed, Kaiser et al. reported that the prevalence of malnourished patients in rehabilitation settings was 50.5%, with 41.2% at risk of malnutrition; only 8.5% were classified as having a normal nutritional status [3]. In addition, malnutrition is associated with longer hospitalizations, higher risk of institutionalization, lower quality of life, and cognitive performance [4,5,6,7]. Patients with malnutrition exhibit poorer performance in activities of daily living (ADL) at admission [8] and poorer recovery of ADL during hospitalization than patients without malnutrition [9]. Thus, malnutrition is a serious condition for patients admitted to a convalescent rehabilitation ward, and one that must be overcome.

Given the negative impact of malnutrition on rehabilitation outcomes, the ability of nutritional indicators to serve as predictors of rehabilitation outcomes has been investigated [10,11,12]. For instance, it was recently reported that three nutritional indicators, the Mini-Nutritional Assessment Short Form (MNA-SF) [13], Geriatric Nutritional Risk Index (GNRI) [14], and Controlling Nutritional Status (CONUT) [15], are good predictors of improved scores on ADL indicators, such as the Functional Independent Measure (FIM) or Barthel Index (BI), in patients admitted to convalescent rehabilitation wards. In addition, we previously reported that nutritional indicators are predictive of rehabilitation effectiveness (REs), which is an indicator of ADL improvement [16,17]. Those studies illustrate the need for a practical nutritional assessment tool capable of predicting functional outcomes with high accuracy. However, there are few studies that have compared nutritional screening tools for prediction of rehabilitation outcomes in patients admitted to convalescent rehabilitation wards.

The aim of this study, therefore, was to determine the nutritional screening tool that optimally associates with rehabilitation outcome among the MNA-SF, GNRI and CONUT in patients admitted to convalescent rehabilitation wards.

## 2. Materials and Methods

### 2.1. Ethics

This study adhered to the Declaration of Helsinki and was approved by the Wakakusa-Tatsuma Rehabilitation Hospital Ethics Committee (approval number: 19100761). The requirement for informed consent was waived because this study was based on a retrospective analysis of routinely collected data. Furthermore, information about the study was disclosed on the bulletin board and hospital homepage, and patients were allowed to refuse use of their medical information through an opt-out procedure.

### 2.2. Participants and Setting

**Figure 1:**
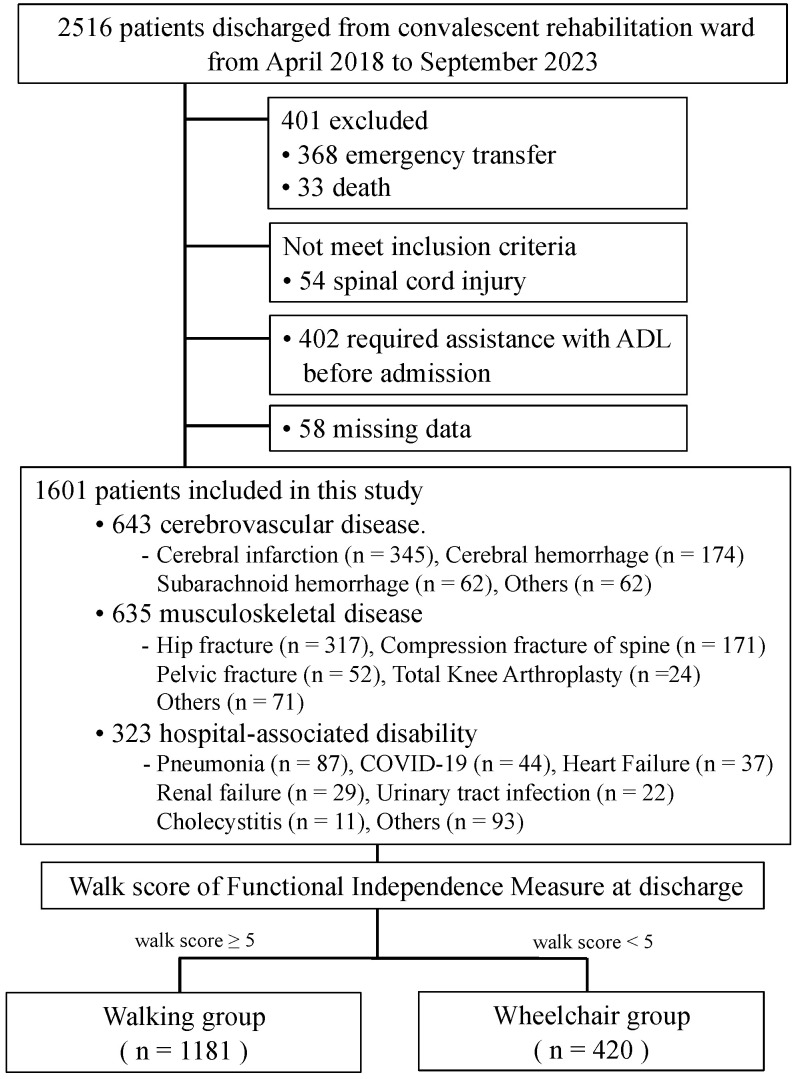
Flow chart of this study.

This retrospective cohort study was conducted in the convalescent rehabilitation wards at Wakakusa-Tatsuma Rehabilitation Hospital in Osaka, Japan. Enrolled in the study were patients admitted to the convalescent rehabilitation wards between April 2018 and September 2023 and subsequently discharged. During the research period, 2516 consecutive patients were admitted. Among those, patients who had a spinal cord injury (n = 54), required emergency transfer (n = 368), died (n = 33), required assistance with ADL before admission (n = 402), or whose data were missing (n = 58) were excluded. Ultimately, 1601 patients were studied (Figure 1). In the convalescent rehabilitation ward in Japan, public medical insurance covers individual rehabilitation for patients provided by physical therapists, occupational therapists, and speech–language–hearing therapists for a maximum of nine units per day (1 unit = 20 min), 7 days per week. The time allocation for each type of rehabilitation can be tailored according to the needs of the individual patient. In this study, patients underwent a rehabilitation program including conventional physical therapy, occupational therapy, and speech–language–hearing therapy which was performed 6 to 8 units per day according to the patient’s condition until discharge. Physical therapists performed active-assisted and active mobilizations, exercises for muscle-strength recovery, postural passages and transfers, sitting-and-standing training, motor-coordination and balance training, and walking training. Patients who were considered to benefit from robot-assisted gait training underwent walking training using the one-leg assisted-gait robot, called Welwalk WW-2000 (Toyota Motor Corporation, Aichi, Japan). Occupational therapists assessed the patient’s home environment and performed ADL training appropriate for the living environment. Speech–language–hearing therapists performed voice training, dysarthria training, cognitive training, and dysphagia rehabilitation.

Basic information, including age, sex, height, weight, body mass index (BMI), reason for admission (e.g., stroke, musculoskeletal disease, or hospitalization-associated disability), comorbidities, onset-to-admission interval, and medications at admission were collected from medical records. Clinical data such as results from physical examinations, swallowing function tests, blood tests and ADL measurements were also collected. The physical examinations included handgrip strength and quadriceps strength. The handgrip strength of the dominant hand (or, in the case of hemiparesis, the non-paralyzed hand) was measured using a Takei TKK 5401 digital dynamometer (Takei Scientific Instruments Co., Ltd., Tokyo, Japan), with the greatest of three measurements recorded. The quadriceps strength of the dominant leg (or, in the case of hemiparesis, the non-paralyzed leg) was measured using a hand-held dynamometer (Sakai Medical Co., Ltd., Tokyo, Japan). For measurement of quadriceps strength, the participant was seated on a plinth, with their back resting against a firm support, thighs fully supported, knees flexed to 90° and the lower legs hanging freely. The pad of a hand-held dynamometer was positioned at 80% of the tibial length, to resist maximal isometric force of the quadriceps. The participants were asked to push against the dynamometer as hard as possible for 3 s. Swallowing function was evaluated using the Functional Oral Intake Scale (FOIS) [18]. Blood tests were performed to evaluate serum albumin, C-reactive protein (CRP), creatinine, estimated glomerular rate (eGFR), and hemoglobin. B-type natriuretic peptide (BNP) was also measured by commercial immunoassay. ADL was evaluated using the BI and FIM instrument. The BI measures ten functions that are important for independent living: eating, dressing, transferring, grooming, bathing, toileting, walking, stair climbing, bowel control, and bladder care. BI scores ranged from 0 to 100 points, with higher BI scores indicating lower dependency [19]. The FIM consists of a motor domain with 13 sub-items and a cognitive domain with 5 sub-items. Each item is scored on a scale of 1 to 7 points. The total FIM-motor and FIM-cognitive scores range from 13 to 91 and 5 to 35 points, respectively. The total FIM scores ranged from 18 (reflecting full dependence) to 126 (reflecting complete independence) [20].

### 2.3. Assessment of the Nutritional Indicators

Each patient’s nutritional status was assessed based on MNA-SF, GNRI and CONUT scores. These nutritional indicators are useful nutritional screening tools that have been validated in several studies of patients admitted to convalescent rehabilitation wards [13,14,15,16,17,21]. The MNA-SF is a simple nutritional screening tool consisting of six questionnaire items (appetite, weight loss, mobility, recent illness/stress, dementia/depression and BMI). MNA-SF scores range from 0 to 14 points, and patients with a score of 12 or less are defined as being at nutritional risk [22]. GNRI scores were calculated from the patients’ BMI and serum albumin concentrations using the following formula: GNRI = (14.89 × serum albumin [g/dL]) + (41.7 × [actual bodyweight/ideal bodyweight]. Ideal body weight was defined as a BMI of 22.0 kg/m^2^. A GNRI < 92 was defined as moderate or severe malnutrition risk, while a GNRI score > 92 was defined as low or no malnutrition risk [23]. CONUT scores were calculated based on serum albumin levels, total peripheral lymphocyte counts, and total cholesterol levels. CONUT scores ranged from 0 to 12 points, and patients with a score of 2 or more were defined as being at nutritional risk [24]. We also collected patients’ energy and protein intakes on admission and discharge. Nutritional indicators were collected or evaluated at the time of admission by a registered dietitian at our hospital. After admission, the registered dietitian evaluated the patient’s nutritional indicators at least once a month, and appropriate nutrition was provided by the registered dietitian in consultation with the patient’s physician, based on the patient’s weight change and the intensity of rehabilitation exercise, and the results were recorded in the patient’s medical record.

### 2.4. Rehabilitation Outcome

The primary rehabilitation outcome was the proportion of patients who were able to walk without assistance at discharge. In this study, a walk FIM score of 5 or higher was defined as the walking group. The detailed scoring criteria for the FIM walk domain indicate that a score of 6 or higher is the ability to walk at least 50 m independently. If a patient has difficulty walking more than 50 m but is able to walk between 15 m and 49 m, the walk FIM score is scored as 5. A walk FIM score of 5 or higher indicates that the patient can walk without assistance, so in a previous report examining the walking patterns of stroke patients, subjects were selected based on a walk FIM score of 5 or higher [25]. We divided the subjects into two groups according to their walking status at discharge: the walking group and the wheelchair group (Figure 1).

As a secondary outcome, we assessed REs, which was calculated using the FIM instrument and the following formula: (FIM at discharge/FIM at admission)/(126 − FIM at admission) × 100%. By expressing REs as a percentage reflecting the proportion of potential improvement actually achieved during rehabilitation, it was corrected for a ceiling effect [26].

### 2.5. Statistical Analysis

Parametric continuous data are presented as the mean ± standard deviation, and non-parametric data as the median (interquartile range 25–75 percentile). Normality was confirmed by the Shapiro–Wilk test. Differences between the two groups defined based on walking ability at discharge were analyzed using Student’s *t*-test or the Mann–Whitney U-test. Differences in REs were evaluated using analysis of covariance (ANCOVA) adjusted for age and gender. Categorical data were expressed as incidences and percentages, and comparisons were made using the chi-square test.

Univariate and multivariate logistic regression analyses were used to determine the association between MNA-SF, GNRI and CONUT scores and the walking status at discharge. In addition, the relationship between each nutritional indicator of nutritional status at admission and walking status at discharge were assessed using receiver operating (ROC) curves. Differences in diagnostic performance were compared based on the area under the ROC curve (AUC). We performed univariate linear regression analysis with REs as a dependent variable. We also performed multiple regression analysis with REs as a dependent variable using factors that showed a significant (*p* < 0.05) correlation with REs in univariate linear regression analysis as independent variables. Multicollinearity between factors was assessed using the variance inflation factor.

Values of *p* < 0.05 were considered statistically significant. Statistical analyses were performed using SPSS version 28.0 (IBM, Armonk, NY, USA).

## 3. Results

Among the 1601 patients studied, the reasons for their hospitalization were stroke in 643 patients, musculoskeletal disease in 635 patients, and hospital-associated disability in 323 patients; 1181 were classified into the walking group and 402 were classified into the wheelchair group. The numbers and percentages of subjects in the independent walking group who were in rehabilitation due to stroke, musculoskeletal disease, and hospital-associated disability were 424 (35.9%), 520 (44.0%) and 237 (20.1%), respectively.

The demographic and clinical baseline characteristics of each group are shown in Table 1. The walking group was significantly younger and had higher BMIs than the wheelchair group; however, there was no significant difference with respect to gender. Regarding comorbidities, the rate of dyslipidemia was significantly higher in the walking group; however, there were no differences in the rates of hypertension, diabetes and atrial fibrillation. Handgrip strength, quadriceps strength and FOIS were all significantly higher in the walking than wheelchair group. ADL parameters at admission, including BI score, motor FIM, cognitive FIM or total FIM score were also significantly higher in the walking than in the wheelchair group. The laboratory data showed that serum albumin and hemoglobin were significantly higher and plasma B-type natriuretic peptide was significantly lower in the walking than in the wheelchair group.

Among the walking group, nutritional status indicated by MNA-SF and GNRI scores were significantly higher and CONUT scores were significantly lower compared to the wheelchair group. Energy intake was also higher in the walking than in the wheelchair group.

Rehabilitation outcomes at discharge among the 1601 subjects are presented in Table 2. Handgrip strength, quadriceps strength, FOIS and ADL parameters were all significantly higher in the walking than in the wheelchair group. REs and rate of discharge to home were also significantly higher in the walking than in the wheelchair group. Length of hospitalization was significantly lower in the walking than in the wheelchair group. In addition, the walking group had significantly higher MNA-SF and GNRI scores and significantly lower CONUT scores than the wheelchair group, with Cohen’s d effect sizes of 0.69, 0.60 and 0.50, respectively (all *p* < 0.01).

Rehabilitation outcomes at discharge for each disease are presented in Table 3. For all diseases, the muscle strength index, FOIS, ADL parameters, and rate of discharge to home were significantly higher in the walking than in the wheelchair group. Length of hospitalization was significantly shorter in the walking than in the wheelchair group for stroke and musculoskeletal diseases, but there was no difference for hospital-associated disability. Among stroke patients, the walking group had significantly higher MNA-SF and GNRI scores and significantly lower CONUT scores than the wheelchair group, with Cohen’s d effect sizes of 0.81, 0.54 and 0.27, respectively (all *p* < 0.01). Likewise, for patients with musculoskeletal diseases, the walking group had significantly higher MNA-SF and GNRI scores and significantly lower CONUT scores than the wheelchair group, with Cohen’s d effect sizes of 0.51, 0.60 and 0.47, respectively (all *p* < 0.01). And for patients with hospital-associated disability, the walking group also had significantly higher MNA-SF and GNRI scores and significantly lower CONUT scores than the wheelchair group, with Cohen’s d effect sizes of 0.66, 0.49 and 0.31, respectively (all *p* < 0.01).

The results of multivariate logistic regression analyses with walking without assistance at discharge as a dependent variable and each nutritional index as an independent variable are shown in Table 4. In the analysis of all 1601 patients, univariate logistic regression showed that MMA-SF, GNRI and CONUT scores as well as age, handgrip strength, quadriceps strength, FOIS, hemoglobin, energy intake and BNP were all significantly associated with walking without assistance at discharge (*p* < 0.001). However, the multivariate logistic regression analysis adjusted for confounders revealed that MNA-SF and GNRI scores were significantly associated with walking without assistance at discharge, but CONUT scores were not. In the analysis of each condition, MNA-SF was independently and significantly associated with walking without assistance at discharge, even after adjusting for confounding factors. Multivariate logistic regression analysis with walking without assistance at discharge as the dependent variable using factors that were significant in the univariate logistic regression analysis as independent variables showed that age, handgrip strength, FOIS, and MNA-SF score were all independently associated with walking without assistance at discharge (all *p* < 0.01).

We also performed ROC analyses to assess walking without assistance at discharge. For each index, the AUC for walking without assistance at discharge was as follows: for the MNA-SF, 0.698 (95% CI: 0.669–0.727); for the GNRI, 0.651 (95% CI: 0.622–0.681); and for the CONUT score, 0.603 (95% CI: 0.572–0.634).

**Figure 2:**
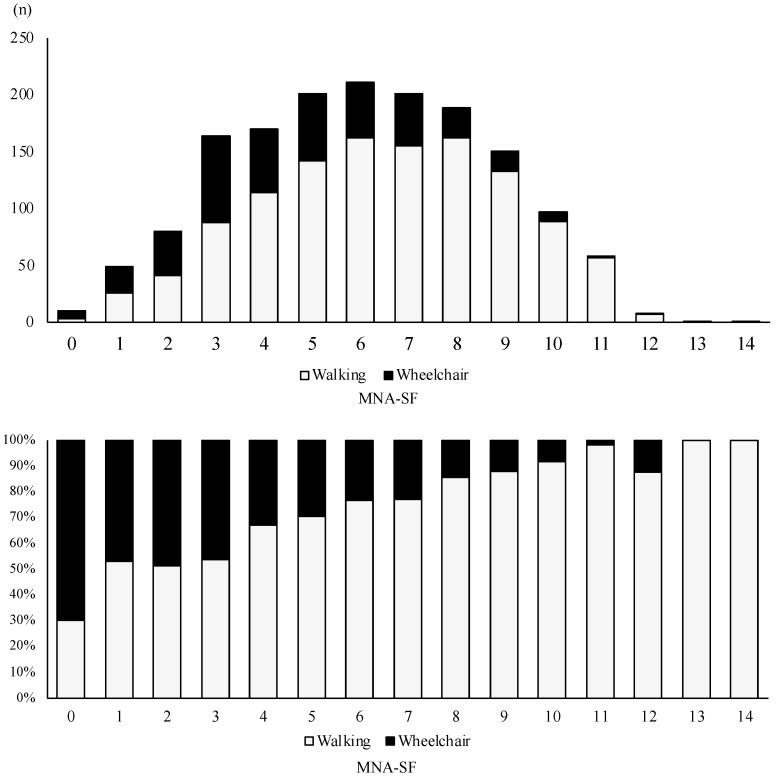
Distribution of patients based on MNA-SF scores at admission and the percentage capable of walking at discharge for each MNA-SF score.

Figure 2 shows the distribution of MNA-SF scores at admission among the patients and the rate of walking without assistance at discharge for each MNA-SF score. Based on the MNA-SF classification, 1096 (68.5%) patients were classified as malnourished, 495 (30.9%) were classified as being at risk of malnutrition, and 10 (0.6%) were classified as well-nourished. The rate of walking without assistance at discharge increased with increases in the MNA-SF scores.

Table 5 shows the results of the multivariate linear regression analysis with REs as the dependent variable and MNA-SF, GNRI and CONUT scores as independent variables. After adjustment for age, sex and other confounding factors, MNA-SF was a significant predictor of REs for patients with stroke or musculoskeletal disease. GNRI scores were significantly associated with REs only for patients with musculoskeletal disease, and CONUT scores were not associated with REs. Multivariate linear regression analysis with REs as the dependent variable using MNA-SF and factors that were significant in the univariate linear regression analysis showed that age, FOIS, handgrip strength, quadriceps strength, and MNA-SF were all independently associated with REs (all *p* < 0.01).

## 4. Discussion

In the present study, we showed that MNA-SF scores were associated with walking without assistance at discharge as a functional outcome across all diseases among patients admitted to convalescent rehabilitation wards, even after adjusting for confounding factors. GNRI and CONUT scores also showed significant associations with walking without assistance at discharge in univariate analyses, but not in a multivariate analysis. In addition, as a secondary outcome, MNA-SF scores were independently associated with REs in patients with stroke and musculoskeletal disease. These results suggest that of the three nutritional indicators tested (MNA-SF, GNRI and CONUT), MNA-SF scores were the best nutritional assessment tool for predicting improvement in physical activity including walking ability and ADL at discharge in patients admitted to convalescent rehabilitation wards.

Many of the patients admitted to convalescent rehabilitation wards are malnourished at admission [2]. In this study, the prevalence of malnutrition assessed with the MNA-SF was 68.5%, which is similar to findings reported previously [2]. MNA-SF scores are used to evaluate elderly patients worldwide, in part because the scoring consists of six items that include functional as well as psychological and cognitive parameters. Additionally, the MNA-SF includes an item that asks about declining food intake and weight loss during the previous 3 months. As a result, a strength point of the MNA-SF is that it can be used to evaluate changes in nutritional indicators over a 3-month period. GNIR and CONUT scores do not give that kind of information.

Patients are admitted to convalescent rehabilitation wards only after completing acute treatment at a hospital. It is therefore possible for weight loss to occur during a patient’s acute hospitalization. Consistent with that idea, Paquereau et al. reported that patients often experience a reduction in body weight of approximately 3 kg during the acute phase of their treatment [27]. Unfortunately, that loss of body weight is reportedly an important feature of malnutrition that can adversely affect a patient’s ability to return to performing ADL [13]. Indeed, BMI at admission is significantly associated with motor FIM gain in stroke patients [28]. Thus, body weight loss prior to admission to a convalescent rehabilitation ward likely influences the progress toward performance of ADL during subsequent rehabilitation. In addition, rehabilitation outcomes of patients with diseases such as stroke, hip fracture, and cardiovascular disease are often negatively affected by cognitive impairment [29,30,31]. All of these factors are addressed by the MNA-SF, making it useful for accurately predicting functional outcomes in elderly patients admitted to convalescent rehabilitation wards [16,17,32,33].

By contrast, our multivariate logistic analysis showed that the GNRI and CONUT were not significantly associated with walking without assistance at discharge. The primary reason is likely the inclusion of serum albumin values in the GNRI and CONUT scores. Serum albumin has traditionally been considered a useful biochemical marker for nutrition assessment [34,35]. However, recent studies have shown that serum albumin reflects inflammation rather than nutritional status or protein–energy malnutrition [36,37]. Both acute and chronic illnesses are characterized by inflammation, and various inflammatory cytokines inhibit the synthesis of albumin, resulting in lower serum albumin concentrations [38]. In addition, inflammation leads to a redistribution of serum proteins due to an increase in capillary permeability and promotes resting energy expenditure, leading to increased protein and energy requirements. Consequently, there is an association between inflammation and serum albumin, but not between malnutrition and serum albumin. By including serum albumin levels, GNRI and CONUT scores may be useful for assessing risk of inflammation-related diseases, but they are not suitable for assessing nutrition per se. In recent years, walking robots have been introduced into rehabilitation aimed at regaining walking ability, and their effectiveness has been reported [39,40]. In addition, because elderly patients may experience age-related intestinal malabsorption [41], robotic rehabilitation based on nutritional assessment may contribute to walking independence at discharge in patients admitted to convalescent rehabilitation wards.

This study has three limitations. First, because it is a retrospective observational study, the causal relationship between baseline malnutrition and outcomes is unclear. Second, it was conducted at a single hospital, so the generalizability of the results may be limited. Multicenter prospective cohort studies will be needed to overcome the limitations of this study. Third, patients were provided with standard rehabilitation treatment in the convalescent rehabilitation wards, but we were unable to mention the details of the type of rehabilitation. We were also unable to collect detailed information about the patients’ social backgrounds. In the future, it will necessary to verify the impact of more detailed rehabilitation factors, including type of rehabilitation provided to patients, contents of rehabilitation programs and patients’ social backgrounds on rehabilitation outcome such as physical activity and discharge to home. Nonetheless, a strength of this study is that we were able to analyze over 1600 consecutive inpatients. From our findings with these patients, we believe that assessing MNA-SF on admission may be useful for predicting improvement in physical activity at discharge in patients admitted to convalescent rehabilitation wards.

## 5. Conclusions

Our findings indicate that of the three nutritional indicators tested (MNA-SF, GNRI and CONUT), MNA-SF scores were the best nutritional assessment tool for predicting improvement in physical activity at discharge in patients admitted to convalescent rehabilitation wards after hospitalization.

## Figures and Tables

**Table 1:** Baseline demographic and clinical characteristics of the walking and the wheelchair groups.

	Total	Walking Group	Wheelchair Group	*p* Value
n	1601	1181	420	
Age, year	77 ± 12	76 ± 13	79 ± 12	<0.001 *
Male, n (%)	739 (46.2)	555 (47.0)	184 (43.8)	0.261 ^†^
Height, m	1.6 ± 0.1	1.6 ± 0.1	1.5 ± 0.1	0.067 *
Weight, kg	51.3 ± 11.9	52.3 ± 12.0	48.5 ± 11.1	<0.001 *
Body mass index, kg/m^2^	21.1 ± 4.0	21.5 ± 4.0	20.3 ± 3.8	<0.001 *
Reason for admission, n (%)				
Stroke	643 (40.2)	424 (35.9)	219 (52.1)	<0.001 ^†^
Musculoskeletal disease	635 (39.7)	520 (44.0)	115 (27.4)	<0.001 ^†^
Hospitalization-associated disability	323 (20.2)	237 (20.1)	86 (20.5)	0.858 ^†^
Comorbidity, n (%)				
Hypertension	997 (62.3)	737 (62.4)	259 (61.7)	0.259 ^†^
Diabetes	368 (23.0)	274 (23.2)	94 (22.4)	0.732 ^†^
Dyslipidemia	346 (21.6)	273 (23.1)	72 (17.1)	0.010 ^†^
Atrial fibrillation	194 (12.1)	133 (11.3)	61 (14.5)	0.078 ^†^
Time from onset, day	29 (21–42)	28 (20–40)	31 (22–46)	0.063 **
Handgrip strength, kg	17.0 ± 10.0	18.1 ± 10.3	12.7 ± 6.6	<0.001 *
Quadriceps strength, kg	13.3 ± 7.3	13.8 ± 7.4	10.7 ± 6.6	<0.001 *
FOIS	6 ± 2	6 ± 1	4 ± 2	<0.001 *
Barthel Index, score	45 (15–65)	50 (35–70)	10 (5–30)	<0.001 **
FIM, score				
Motor	32 (20–45)	38 (25–48)	16 (13–23)	<0.001 **
Cognitive	20 (14–25)	23 (18–27)	13 (8–17)	<0.001 **
Total	52 (36–69)	60 (45–74)	31 (22–39)	<0.001 **
Nutritional Index at admission				
MNA-SF	6.1 ± 2.6	6.5 ± 2.5	4.7 ± 2.4	<0.001 *
GNRI	91.4 ± 12.4	93.1 ± 12.4	86.7 ± 10.9	<0.001 *
CONUT score	3.3 ± 2.3	3.1 ± 2.3	3.9 ± 2.3	<0.001 *
Energy intake, kcal	1405 ± 406	1453 ± 394	1270 ± 407	<0.001 *
Protein intake, g	56.7 ± 15.7	58.4 ± 15.1	51.9 ± 16.4	<0.001 *
Medication, n	5.2 ± 3.0	5.2 ± 3.1	5.1 ± 2.8	0.402 *
BNP, pg/mL	37 (17–86)	35 (16–80)	43 (18–100)	<0.001 **
Albumin, g/dL	3.5 ± 0.5	3.5 ± 0.5	3.2 ± 0.5	<0.001 *
CRP, mg/dL	0.9 ± 1.8	0.8 ± 1.7	1.1 ± 2.0	0.002 *
Creatinine, mg/dL	0.9 ± 0.4	0.9 ± 0.4	0.8 ± 0.4	0.043 *
eGFR, mg/min	63.8 ± 19.0	63.6 ± 18.7	64.3 ± 20.1	0.002 *
Hemoglobin, g/dL	12.0 ± 1.8	12.1 ± 1.8	11.7 ± 1.8	<0.001 *

Data are presented as mean ± SD for parametric continuous data, median (IQT) for non-parametric data, and n (%) for categorical data. * student’s *t* test. ** Mann–Whitney U test. ^†^ chi-square test. BNP: B-type natriuretic peptide, CONUT: Controlling Nutritional Status, CRP: C-reactive protein, eGFR: estimated glomerular filtration rate, FIM: Functional Independence Measure, FOIS: Functional Oral Intake Scale, GNRI: Geriatric Nutritional Risk Index, MNA-SF: Mini Nutritional Assessment-Short Form.

**Table 2:** Rehabilitation outcomes of the Walking and Wheelchair groups.

	Total	Walking Group	Wheelchair Group	*p* Value
n	1601	1181	420	
Handgrip strength, kg	17.6 ± 8.4	18.8 ± 8.4	12.9 ± 6.9	<0.001 *
Quadriceps strength, kg	15.7 ± 7.8	16.6 ± 7.9	11.4 ± 5.9	<0.001 *
FOIS	6 ± 2	7 ± 1	4 ± 2	<0.001 *
Barthel Index, score	85 (55–100)	90 (85–100)	35 (15–55)	<0.001 **
FIM, score				
Motor	73 (51–83)	79 (70–86)	31 (19–44)	<0.001 **
Cognitive	25 (19–31)	28 (23–33)	16 (12–21)	<0.001 **
Total	98 (71–114)	107 (95–117)	50 (32–64)	<0.001 **
Nutritional Index at discharge				
MNA-SF	9.4 ± 2.8	9.9 ± 2.6	8.1 ± 2.8	<0.001 *
GNRI	89.9 ± 12.3	92.0 ± 11.8	84.9 ± 12.1	<0.001 *
CONUT	3.1 ± 2.7	2.7 ± 2.2	3.9 ± 2.6	<0.001 *
Energy intake, kcal	1582 ± 354	1622 ± 334	1469 ± 382	<0.001 *
Protein intake, g	63.7 ± 14.4	65.2 ± 13.1	59.5 ± 16.8	<0.001 *
Handgrip strength gain, kg	0.7 (0.0–2.7)	0.9 (0.0–2.8)	0.5 (0.0–2.4)	0.624 **
Quadriceps strength gain, kg	1.8 (0.0–4.5)	1.9 (0.2–4.7)	0.8 (0.0–0.3)	<0.001 **
Barthel Index gain, score	30 (15–45)	35 (20–50)	20 (5–35)	<0.001 **
FIM gain, score				
Motor	34 (19–43)	39 (30–46)	12 (4–21)	<0.001 **
Cognitive	4 (1–7)	5 (1–8)	2 (1–5)	<0.001 **
Total	38 (22–50)	43 (33–53)	14 (6–25)	<0.001 **
Rehabilitation effectiveness, %	55.9 ± 38.3	68.9 ± 35.5	19.2 ± 15.2	<0.001 ^§^
Length of hospital stay, day	86 (64–117)	85 (60–93)	90 (81–165)	<0.001 **
Discharge to home, n (%)	1033 (64.5)	876 (74.2)	156 (37.1)	<0.001 ^†^

Data are presented as mean ± SD for parametric continuous data, median (IQT) for non-parametric data, and n (%) for categorical data. * student’s *t* test. ** Mann–Whitney U test. ^†^ chi-square test. ^§^ analysis of covariance adjusted for age and gender. CONUT: Controlling Nutritional Status, FIM: Functional Independence Measure, FOIS: Functional Oral Intake Scale, GNRI: Geriatric Nutritional Risk Index, MNA-SF: Mini Nutritional Assessment-Short Form.

**Table 3:** Rehabilitation outcomes for stroke, musculoskeletal diseases, and hospital-associated disability.

	Total	Walking Group	Wheelchair Group	*p* Value
Stroke				
n	643	424	219	
Handgrip strength at admission, kg	18.4 ± 12.5	20.0 ± 13.3	13.4 ± 7.4	<0.001 *
Quadriceps strength at admission, kg	14.3 ± 7.9	15.0 ± 7.8	10.9 ± 7.2	<0.001 *
FOIS at admission	5 ± 2	6 ± 2	4 ± 2	<0.001 *
Barthel Index at admission, score	38 (10–60)	50 (30–70)	10 (0–20)	<0.001 **
FIM at admission, score				
Motor	26 (15–42)	36 (23–47)	14 (13–19)	<0.001 **
Cognitive	18 (11–23)	21 (16–25)	10 (7–16)	<0.001 **
Total	44 (28–64)	58 (42–71)	25 (21–34)	<0.001 **
Nutritional Index at admission				
MNA-SF	5.7 ± 2.6	6.4 ± 2.5	4.4 ± 2.3	<0.001 *
GNRI	92.6 ± 12.5	94.8 ± 12.5	88.2 ± 11.4	<0.001 *
CONUT	3.2 ± 2.3	3.0 ± 2.2	3.7 ± 2.3	<0.001 *
Energy intake, kcal	1435 ± 417	1522 ± 406	1265 ± 388	<0.001 *
Protein intake, g	57.7 ± 15.7	60.7 ± 15.0	51.9 ± 15.6	<0.001 *
Handgrip strength at discharge, kg	19.0 ± 9.1	21.0 ± 9.0	13.7 ± 3.7	<0.001 *
Quadriceps strength at discharge, kg	16.8 ± 8.5	18.3 ± 8.5	11.2 ± 5.6	<0.001 *
FOIS at discharge	6 ± 2	7 ± 1	4 ± 2	<0.001 *
Barthel Index at discharge, score	85 (45–100)	95 (85–100)	30 (10–50)	<0.001 **
FIM at discharge, score				
Motor	70 (37–84)	80 (70–87)	29 (17–41)	<0.001 **
Cognitive	24 (17–30)	28 (23–32)	15 (10–20)	<0.001 **
Total	94 (55–112)	108 (95–117)	44 (31–59)	<0.001 **
Nutritional Index at discharge				
MNA-SF	9.2 ± 2.9	10.0 ± 2.6	7.8 ± 2.8	<0.001 *
GNRI	91.6 ± 12.2	94.9 ± 10.0	86.5 ± 13.5	<0.001 *
CONUT	2.8 ± 2.2	2.4 ± 1.9	3.4 ± 2.5	<0.001 *
Energy intake, kcal	1269 ± 377	1704 ± 337	1482 ± 408	<0.001 *
Protein intake, g	65.2 ± 15.2	67.9 ± 12.8	60.0 ± 17.9	<0.001 *
Handgrip strength gain, kg	1.1 (0.0–3.3)	1.2 (0.0–3.6)	1.0 (0.0–3.2)	0.791 **
Quadriceps strength gain, kg	2.0 (0.2–5.0)	2.3 (0.4–5.2)	1.0 (0.0–4.0)	0.022 **
Barthel Index gain, score	30 (15–45)	35 (20–50)	18 (5–30)	<0.001 **
FIM gain, score				
Motor	33 (15–44)	41 (31–49)	11 (3–20)	<0.001 **
Cognitive	5 (2–9)	6 (3–10)	3 (1–6)	<0.001 **
Total	38 (19–52)	46 (35–57)	15 (5–26)	<0.001 **
Rehabilitation effectiveness, %	51.8 ± 30.3	69.0 ± 20.1	18.5 ± 15.5	<0.001 ^§^
Length of hospital stay, day	141 (89–176)	129 (76–173)	161 (114–178)	<0.001 **
Discharge to home, n (%)	361 (56.1)	299 (70.5)	62 (28.3)	<0.001 ^†^
Musculoskeletal disease,				
n	635	520	115	
Handgrip strength at admission, kg	16.5 ± 8.3	17.2 ± 8.4	12.3 ± 6.0	<0.001 *
Quadriceps strength at admission, kg	12.7 ± 6.9	13.0 ± 6.9	11.3 ± 6.4	0.063 *
FOIS at admission	6 ± 1	6 ± 1	5 ± 2	<0.001 *
Barthel Index at admission, score	50 (30–70)	55 (40–70)	20 (5–38)	<0.001 **
FIM at admission, score				
Motor	36 (24–48)	40 (29–49)	22 (15–27)	<0.001 **
Cognitive	23 (17–28)	25 (20–28)	15 (11–19)	<0.001 **
Total	59 (42–75)	64 (50–78)	38 (27–45)	<0.001 **
Nutritional Index at admission				
MNA-SF	6.7 ± 2.5	7.0 ± 2.5	5.7 ± 2.4	<0.001 *
GNRI	92.6 ± 11.6	93.8 ± 11.7	87.0 ± 9.2	<0.001 *
CONUT	3.1 ± 2.1	2.9 ± 2.1	3.9 ± 2.2	<0.001 *
Energy intake, kcal	1390 ± 379	1406 ± 371	1316 ± 408	0.031 *
Protein intake, g	56.2 ± 14.9	56.9 ± 14.7	53.1 ± 15.9	0.019 *
Handgrip strength at discharge, kg	16.5 ± 7.9	17.4 ± 8.0	12.8 ± 5.7	<0.001 *
Quadriceps strength at discharge, kg	15.1 ± 7.3	15.6 ± 7.3	12.0 ± 6.5	<0.001 *
FOIS at discharge	6 ± 1	7 ± 1	5 ± 2	<0.001 *
Barthel Index at discharge, score	90 (70–100)	95 (85–100)	50 (23–60)	<0.001 **
FIM at discharge, score				
Motor	76 (62–84)	79 (70–80)	40 (27–52)	<0.001 **
Cognitive	27 (21–33)	29 (24–34)	17 (13–22)	<0.001 **
Total	103 (83–116)	107 (95–118)	57 (42–72)	<0.001 **
Nutritional Index at discharge				
MNA-SF	9.8 ± 2.5	10.0 ± 2.5	9.0 ± 2.5	<0.001 *
GNRI	89.9 ± 12.2	90.9 ± 12.5	85.4 ± 9.7	<0.001 *
CONUT	3.1 ± 2.4	2.9 ± 2.3	4.0 ± 2.6	0.005 *
Energy intake, kcal	1535 ± 303	1546 ± 303	1481 ± 301	0.037 *
Protein intake, g	62.4 ± 12.3	63.1 ± 12.1	59.6 ± 12.8	<0.001 *
Handgrip strength gain, kg	0.5 (0.0–2.2)	0.5 (0.0–2.3)	0.3 (0.0–1.6)	0.254 **
Quadriceps strength gain, kg	1.6 (0.0–3.9)	1.6 (0.1–4.1)	1.1 (0.0–2.1)	0.005 **
Barthel Index gain, score	30 (20–45)	35 (20–45)	20 (5–25)	<0.001 **
FIM gain, score				
Motor	34 (23–43)	37 (29–44)	15 (7–25)	<0.001 **
Cognitive	3 (1–6)	3 (1–7)	2 (1–5)	<0.001 **
Total	38 (26–47)	41 (31–50)	18 (9–30)	<0.001 **
Rehabilitation effectiveness, %	61.6 ± 48.0	70.3 ± 48.3	22.2 ± 15.5	<0.001 ^§^
Length of hospital stay, day	78 (56–87)	75 (53–86)	85 (71–89)	<0.001 **
Discharge to home, n (%)	475 (74.8)	411 (79.0)	64 (55.7)	<0.001 ^†^
Hospitalization-associated disability,				
n	323	237	86	
Handgrip strength at admission, kg	15.7 ± 7.3	16.7 ± 7.3	11.9 ± 5.8	<0.001 *
Quadriceps strength at admission, kg	12.6 ± 7.1	13.3 ± 7.3	9.6 ± 5.5	<0.001 *
FOIS at admission	5 ± 2	6 ± 2	4 ± 2	<0.001 *
Barthel Index at admission, score	40 (20–60)	50 (35–65)	13 (5–30)	<0.001 **
FIM at admission, score				
Motor	28 (19–41)	35 (24–45)	17 (13–24)	<0.001 **
Cognitive	20 (15–25)	22 (17–27)	15 (9–21)	<0.001 **
Total	49 (37–60)	56 (44–71)	35 (25–43)	<0.001 **
Nutritional Index at admission				
MNA-SF	5.5 ± 2.4	5.9 ± 2.4	4.3 ± 2.2	<0.001 *
GNRI	86.7 ± 12.5	88.3 ± 12.8	82.4 ± 10.5	<0.001 *
CONUT	4.0 ± 2.6	3.8 ± 2.6	4.6 ± 2.4	0.009 *
Energy intake, kcal	1375 ± 429	1433 ± 406	1218 ± 450	<0.001 *
Protein intake, g	55.6 ± 16.9	54.7 ± 15.7	50.4 ± 19.0	0.003 *
Handgrip strength at discharge, kg	16.8 ± 7.5	17.9 ± 7.2	12.7 ± 7.2	<0.001 *
Quadriceps strength at discharge, kg	14.7 ± 7.3	15.6 ± 7.4	11.1 ± 5.7	<0.001 *
FOIS at discharge	6 ± 2	6 ± 1	4 ± 2	<0.001 *
Barthel Index at discharge, score	85 (55–95)	90 (80–100)	35 (10–55)	<0.001 **
FIM at discharge, score				
Motor	71 (47–81)	77 (67–84)	31 (17–41)	<0.001 **
Cognitive	25 (19–31)	28 (23–32)	18 (14–24)	<0.001 **
Total	97 (68–110)	103 (91–114)	51 (32–64)	<0.001 **
Nutritional Index at discharge				
MNA-SF	9.0 ± 2.8	9.5 ± 2.7	7.6 ± 2.7	<0.001 *
GNRI	87.1 ± 12.2	89.8 ± 12.1	81.0 ± 10.0	<0.001 *
CONUT	3.6 ± 2.5	3.0 ± 2.2	4.8 ± 2.6	<0.001 *
Energy intake, kcal	1583 ± 386	1641 ± 360	1422 ± 412	<0.001 *
Protein intake, g	63.2 ± 16.3	65.0 ± 15.0	58.2 ± 18.5	0.003 *
Handgrip strength gain, kg	1.0 (0.0–2.5)	1.1 (0.0–3.0)	0.3 (0.0–2.1)	0.086 **
Quadriceps strength gain, kg	1.4 (0.0–4.7)	1.6 (0.1–4.8)	0.8 (0.0–2.4)	0.049 **
Barthel Index gain, score	35 (15–45)	40 (25–50)	15 (5–34)	<0.001 **
FIM gain, score				
Motor	34 (19–44)	39 (31–47)	10 (3–18)	<0.001 **
Cognitive	3 (1–7)	4 (1–8)	2 (1–6)	0.006 **
Total	38 (21–50)	43 (34–54)	14 (6–22)	<0.001 **
Rehabilitation effectiveness, %	52.9 ± 28.7	65.8 ± 20.5	17.1 ± 13.5	<0.001 ^§^
Length of hospital stay, day	85 (62–89)	85 (62–88)	86 (71–90)	0.702 **
Discharge to home, n (%)	197 (61.0)	167 (70.5)	30 (34.9)	<0.001 ^†^

Data are presented as mean ± SD for parametric continuous data, median (IQT) for non-parametric data, and n (%) for categorical data. * student’s *t* test. ** Mann–Whitney U test. ^†^ chi-square test. ^§^ analysis of covariance adjusted for age and gender. CONUT: Controlling Nutritional Status, FIM: Functional Independence Measure, GNRI: Geriatric Nutritional Risk Index, MNA-SF: Mini Nutritional Assessment-Short Form.

**Table 4:** Univariate and multivariate logistic regression analyses of the association between each nutritional screening tool and walking without assistance at discharge.

	Crude Model			Adjusted Model 1			Adjusted Model 2		
	OR	95% CI	*p* Value	OR	95% CI	*p* Value	OR	95% CI	*p* Value
Overall									
MNA-SF	1.336	1.272–1.404	<0.0001	1.961	1.961–1.399	<0.0001	1.185	1.185–1.292	<0.0001
GNRI	1.045	1.035–1.056	<0.0001	1.043	1.032–1.054	<0.0001	1.021	1.001–1.042	0.038
CONUT	0.886	0.826–0.908	<0.0001	0.882	0.839–0.927	<0.0001	0.941	0.857–1.033	0.199
Stroke									
MNA-SF	1.386	1.286–1.494	<0.0001	1.366	1.266–1.474	<0.0001	1.163	1.011–1.338	0.035
GNRI	1.046	1.031–1.601	<0.0001	1.039	1.023–1.055	<0.0001	0.989	0.959–1.020	0.484
CONUT	0.887	0.827–0.953	0.001	0.929	0.861–1.001	0.054	1.155	0.998–1.337	0.053
Musculoskeletal disease									
MNA-SF	1.222	1.126–1.327	<0.0001	1.188	1.091–1.293	<0.0001	1.182	1.037–1.347	0.012
GNRI	1.053	1.034–1.073	<0.0001	1.047	1.027–1.068	<0.0001	1.027	0.999–1.056	0.057
CONUT	0.813	0.742–0.891	<0.0001	0.836	0.758–0.921	<0.0001	0.925	0.809–1.057	0.251
Hospital-associated disability									
MNA-SF	1.474	1.291–1.684	<0.0001	1.501	1.305–1.726	<0.0001	1.318	1.053–1.650	0.016
GNRI	1.044	1.021–1.068	<0.0001	1.042	1.018–1.067	0.001	1.030	0.998–1.073	0.163
CONUT	0.896	0.813–0.998	0.027 *	0.911	0.823–1.009	0.073	0.851	0.719–1.009	0.063

CONUT: Controlling Nutritional Status, GNRI: Geriatric Nutritional Risk Index, MNA-SF: Mini Nutritional Assessment-Short Form. Adjusted Model 1: adjusted for age and sex. Adjusted Model 2: adjusted for age and sex as well as variables with a *p*-value of <0.05 in the univariate analyses (handgrip strength, quadriceps strength, Functional Oral Intake Scale, energy intake, B-type natriuretic peptide, and hemoglobin). * Body mass index was excluded because it is a component of the GNRI and MNA-SF.

**Table 5:** Univariate and multivariate liner regression analyses of the association between each nutritional screening tool and rehabilitation effectiveness.

	Crude Model				Adjusted Model 1				Adjusted Model 2			
	β	B	95% CI	*p* Value	β	B	95% CI	*p* Value	β	B	95% CI	*p* Value
Overall												
MNA-SF	0.257	0.038	0.031–0.045	<0.0001	0.239	0.035	0.028–0.042	<0.0001	0.128	0.012	0.006–0.019	<0.0001
GNRI	0.231	0.007	0.006–0.009	<0.0001	0.198	0.006	0.005–0.009	<0.0001	0.091	0.002	0.001–0.003	0.004
CONUT	−0.14	−0.023	−0.031–−0.015	<0.0001	−0.105	−0.017	−0.026–−0.009	<0.0001	−0.058	−0.006	−0.013–0.001	0.052
Stroke												
MNA-SF	0.404	0.046	0.038–0.054	<0.0001	0.365	0.042	0.034–0.050	<0.0001	0.155	0.016	0.005–0.026	0.004
GNRI	0.351	0.008	0.007–0.010	<0.0001	0.292	0.007	0.005–0.009	<0.0001	0.098	0.002	0.001–0.004	0.067
CONUT	−0.22	−0.029	−0.039–−0.019	<0.0001	−0.155	−0.021	−0.031–−0.010	<0.0001	−0.013	−0.002	−0.013–0.010	0.792
Musculoskeletal disease												
MNA-SF	0.136	0.026	0.011–0.041	<0.0001	0.098	0.019	0.004–0.033	0.014	0.108	0.01	0.001–0.019	0.026
GNRI	0.174	0.007	0.004–0.010	<0.0001	0.133	0.006	0.002–0.009	0.001	0.114	0.002	0.001–0.004	0.02
CONUT	−0.086	−0.019	−0.037–−0.02	0.031	−0.05	−0.011	−0.029–0.007	0.218	−0.067	−0.007	−0.017–0.003	0.149
Hospital-associated disability												
MNA-SF	0.32	0.039	0.026–0.052	<0.0001	0.296	0.036	0.024–0.049	<0.0001	0.082	0.009	0.006–0.024	0.253
GNRI	0.196	0.004	0.002–0.007	<0.0001	0.149	0.003	0.001–0.006	0.008	−0.036	−0.001	0.003–0.002	0.587
CONUT	−0.12	−0.014	−0.026–−0.001	0.031	−0.073	−0.008	−0.021–0.004	0.194	−0.029	−0.003	−0.015–0.009	0.634

CONUT: Controlling Nutritional Status, GNRI: Geriatric Nutritional Risk Index, MNA-SF: Mini Nutritional Assessment-Short Form. Adjusted Model 1: adjusted for age and sex. Adjusted Model 2: adjusted for age and sex as well as variables with a *p*-value of <0.05 in the univariate analyses (handgrip strength, quadriceps strength, Functional Oral Intake Scale, and energy intake).

## Data Availability

The data are not publicly available, owing to opt-out restrictions. Data sharing is not applicable.
